# The glia-neutrophil axis: an understudied crosstalk in bacteria-induced neuroinflammation

**DOI:** 10.3389/fneur.2026.1765892

**Published:** 2026-03-11

**Authors:** Andrew M. Dunphy, Ian Marriott

**Affiliations:** Department of Biological Sciences, University of North Carolina at Charlotte, Charlotte, NC, United States

**Keywords:** astrocytes, bacteria, CNS, inflammation, microglia, neutrophils

## Abstract

Bacterial infections of the central nervous system (CNS) are characterized by rapid and devastating neuroinflammation. While inflammation plays an important physiological role in defense against bacteria, such responses within the confines of the cranium can be lethal. Glial cells, including microglia and astrocytes, can perceive bacteria or their products and then respond in a manner that can promote inflammation, changes to blood–brain barrier integrity, and recruit leukocytes into the CNS. In this review, we have summarized their ability to produce chemotactic factors in response to bacterial components and clinically relevant bacterial pathogens of the CNS. Importantly, we have highlighted the fact that the chemotactic factors produced by bacterially challenged glia tend to preferentially recruit neutrophils, and we have described how such cells could then respond to the presence of bacteria to further promote glial activation and their own recruitment. This then, could form a vicious cycle that precipitates the rapid inflammatory CNS damage associated with bacterial infection. However, it is also becoming apparent that glia, and perhaps neutrophils, can adjust their responses to bacteria temporally in such a way as to break this positive feedback loop, and we have described the available evidence for the delayed production for anti-inflammatory mediators by these cells following challenge. Finally, we have discussed the present limitations in our understanding of these cell–cell interactions and their study that must be overcome before we can manipulate such a glia-neutrophil axis for therapeutic purposes.

## 1 Introduction

Bacteria-induced inflammatory diseases, including meningitis and encephalitis, represent a distinct subset of central nervous system (CNS) disorders that are characterized by rapid and devastating innate immune-driven inflammatory responses rather than adaptive immunity (1). Multiple bacterial species have been reported to elicit such lethal CNS inflammation, but *Neisseria meningitidis* and *Streptococcus pneumoniae* remain the most common causative agents (2), while *Listeria monocytogenes* appears to be the deadliest with a 27% mortality rate reported in a recent 80-year period systematic review and meta-analysis (3). Other notable bacterial agents that can elicit profound CNS inflammation include *Borrelia burgdorferi*, the Gram-negative spirochete responsible for Lyme neuroborreliosis that can manifest as meningitis, cranial neuritis, or radiculoneuritis (4), and *Haemophilus influenzae*, *Mycobacterium tuberculosis*, *Escherichia coli*, *Brucella abortus*, *Pseudomonas aeruginosa*, and *Staphylococcus aureus* (3, 5–7).

While bacterial meningitis is typically associated with infection of the meninges and the associated activation of immune cells outside of the CNS, the bacteria that infect the brain parenchyma must be capable of breaching the anatomical barriers that surround the brain, which include the blood-cerebrospinal fluid barrier (BSCFB) and the blood–brain barrier (BBB). For example, *N. meningiditis* is a human-specific Gram-negative bacterium that commensally colonizes the nasopharynx, but some serogroups can cross the BCSFB and infiltrate the CNS, perhaps via transmigration through choroid epithelial cells (8, 9). *S. pneumoniae* is also an Gram-positive commensal of the upper respiratory tract and is similarly thought to translocate across CNS barriers, although the exact location of such translocation remains a point of contention (10–13). This organism is responsible for 58% of such CNS infections, while Group B streptococcus, *H. influenzae*, and *N. meningitidis* are responsible for approximately 21, 11, and 7% of cases, respectively (14).

Following entry into the brain parenchyma, bacteria including *S. pneumoniae* can elicit direct damage via the release of toxins that trigger CNS cell death by apoptosis and severe acute inflammation (15, 16). *S. pneumoniae* CNS infections lead to death in over 20% of cases and lifelong sequelae in approximately 50% of those who survive, and these bacteria-induced long-term CNS effects can include hearing loss, blindness, epilepsy, memory and learning deficits, and stroke (16). The incidence of bacterial meningitis is highest in infants aged 0–2 months (14) and nearly 30% of subjects who experienced childhood meningitis were found to subsequently develop at least one disability, most frequently, emotional disorders, hearing loss, or visual disturbances (17). Similarly, 32% of bacterial meningitis survivors have been reported to demonstrate cognitive impairment, with higher susceptibility if pneumococcal in nature (18).

Importantly, these consequences can occur despite vaccination and/or antibiotic administration, and these treatments often fail to restore CNS sterility. Furthermore, they can also serve as the trigger for longer term physiological complications (15, 16). These consequences are due, in large part, to the sensitivity of the brain to inflammation (19). While inflammation is an important physiological mechanism to combat bacterial infections and initiate repair, bacteria-induced inflammation within the anatomically enclosed compartment of the CNS can have devastating consequences. In the case of *S. pneumoniae*, bacteria-induced inflammation compromises BBB integrity (20) resulting in increased intracranial pressure, potentially leading to hydrocephalus, ischemia, and pleocytosis (21, 22), and changes in the neuronal microenvironment that can lead to demyelination and axonal injury (23).

Bacteria-induced systemic inflammation can compromise the BBB allowing more bacteria and inflammatory stimuli to enter brain tissue (23), but the vulnerability of the CNS is also driven by the rapid recruitment of neutrophils across the BBB (15, 24). Like inflammation, the recruitment of neutrophils is a common consequence of localized infection throughout the body and plays a critical role in host defense. Upon bacterial clearance, these effector cells are subsequently eliminated via apoptosis, which serves to dismantle inflammation and paves the way for tissue repair (25, 26). Indeed, the 89% survival rate reported in a recent meta-analysis of bacterial meningitis cases (14) points to the effectiveness of such defenses in combination with appropriate clinical care. However, excessive neutrophil recruitment and/or activity can further exacerbate inflammatory CNS damage. Neutrophil-associated CNS pathology is due to their release of multiple damaging factors and the more recently described neutrophil extracellular traps (NETs) extensively reviewed elsewhere (27–29), and the fact that the CNS is uniquely populated with specialized glial cells that are highly reactive to pathogens and inflammatory stimuli (19, 30). These cells, including microglia and astrocytes, have long been known for their homeostatic functions within the brain, but they have more recently been recognized to play a key role in neuroinflammation (31–34).

In the present review, we discuss the growing body of evidence that resident CNS cells can perceive bacterial pathogens via an array of microbial pattern recognition receptors (PRRs) to trigger the release of soluble chemotactic mediators that actively promote neutrophil recruitment. Furthermore, upon entering the CNS and subsequent activation these cells can then respond to augment glial immune functions and both directly and indirectly promote further neutrophil recruitment, and we discuss the available evidence for such a vicious cycle and focus on its potential detrimental consequences in both acute and chronic CNS disorders.

## 2 Glial cells recognize bacteria to impact BBB function and promote leukocyte recruitment

The ability of CNS cells, such as microglia and astrocytes, to respond to bacterial pathogens is now well established (31, 32, 35, 36). The means by which these responses are initiated has become apparent with the description of an array of cell surface and cytosolic PRRs for bacterial pathogen associated molecular patterns (PAMPs), as summarized in Table 1. Such PRRs have been broadly classified into five major groups, the Toll-like receptors (TLRs), the nucleotide binding oligomerization domain-containing (NOD)-like receptors (NLRs), the retinoic acid-inducible gene I (RIG-I)-like receptors (RLRs), the C-type lectin receptors (CLRs), and the absent in melanoma-2 (AIM2)-like receptors (ALRs) (37).

**Table 1:** Pattern recognition receptors (PRRs) expressed by glia for clinically relevant bacteria and/or their components.

Cell type	Organism	PRR	Constitutive or inducible	Bacteria or component	Stimulus: Response
Microglia	Human	TLR1 (33, 39)	C (33)/ I, *Bb* (39)		***Bb***: ↑ MHCII, CD11c, IL-10, TNF (39)
		TLR2 (33, 39)	C (33)/ I, *Bb* (39)	PGN (50)	
		TLR3 (33)	C (33)		
		TLR4 (33)	C (28)/ I, LPS, FlaB3 (40)	LPS (40, 50), FlaB3 (40)	**LPS**: ↑ IL-6, CXCL8 (40) **FlaB3: ↑** IL-1β, IL-6, TNF, CXCL8 (40)
		TLR5 (33)	C (33)		
		TLR6 (33)	C (33)		
		TLR7 (33)	C (33)		
		TLR8 (33)	C (33)		
		TLR9 (33)	C (33)	CpG (50)	
		RIG-I (52)	C (52) / I, *Nm, Sa,* 5’-pppRNA (52)	5’-pppRNA (52)	**5’-pppRNA**: ↑ IFN-β (52)
		cGAS (53)	C (53)		
	Murine	TLR1 (50)	C (50)		
		TLR2 (50, 96)	C (50, 96) /I, LPS, *Ss* (50, 96)	PGN (50) *Ba* (83)	**PGN**: ↑ IFN-β, IL-1β, IL-6, IL-10, IL-12, IL-18, TNF, iNOS, CCL3, CD11b, CD45, CD80, CD86, ICAM-1, MHCI (50) **Ba:** ↑ IL-1b (83)
		TLR3 (50)	C (50)		
		TLR4 (50)	C (50) / LPS (50)	LPS (41)	**LPS**: ↑ IFN-α/β, IL-1β, IL-2, IL-6, IL-10, IL-12, TNF, iNOS, CCL2, CCL3, CCL5, GMCSF, CD11b, CD40, CD45, CD80, CD86, ICAM1, MHCI, MHCII (50)
		TLR5 (50)	C (50)	FLG (42)	**FLG**: ↑ IL-6, TNF (42, 134)
		TLR6 (50)	C (50) / I, LPS (50)		
		TLR7 (50)	C (50)		
		TLR8 (50)	C (50) / I, LPS (50)		
		TLR9 (50)	C (50) / I, LPS, CpG (50)	CpG (50)	**CpG**: ↑ IFN-β, IL-1β, IL-2, IL-6, IL-10, IL-12, IL-18, iNOS, GMCSF, CD11b, CD40, CD80, CD86, ICAM-1, MHCI, MHCII (50)
		NOD1 (46)	C (46)		
		NOD2 (46)	C (46) / I, *Nm*, *Ss* (46, 48)	MDP (46)	**MDP**: ↑ IL-1β (46)**MDP + LPS/FLG:** ↑ IL-1β, IL-6, TNF (46)
		NLRP1 (58)	C (58)		
		NLRP3 (58)	C (58)	*Ba* (83)	***Ba***: ↑ IL-1β (83)
		NAIP (58)	C (58)		
		NLRC4 (58)	C (58)		
		RIG-I (51, 52)	C (51, 52) / I, *Nm*, *Sa*, *Sp*, LPS, FLG (52)		
		MDA5 (51)	C (51)		
		LGP2 (51)	C (51)		
		cGAS (54, 57)	C (57)		
		AIM2 (57, 58)	C (57, 58)	*Ba* (83)	***Ba***: ↑ IL-1β (83)
		Mincle (49)	C (49) / I, LPS (49)		
Astrocytes	Human	TLR1 (43, 122)	C (43, 122), I, LPS (122)		
		TLR2 (122)	C (33, 122) / I, LPS (122)		
		TLR3 (22)	C (33, 122) / I (122)		
		TLR4 (122)	C (122)	LPS (122)	
		TLR5 (44)	C (44)	FLG (72)	**FLG: ↑** IL-6 (72)
		TLR9 (44)	C (44)		
		NLRP2 (59)	C (59)		
		NLRP3 (59)	C (59)		
		RIG-1 (52)	C (52) / I, *Sa* (52)		
		MDA5 (62)			
		cGAS (53)	C (53)		
		AIM2 (59)	C (59)		
	Murine	TLR2 (38)	C (38) / I, PGN, LPS, FLG, CpG (38)	PGN (38)	***Ba***: ↑ TNF, IL-1β (83)
		TLR3 (45)	C (45)		
		TLR4 (38)	C (38)	LPS (38)	**LPS**: ↑ CXCL10 (129)
		TLR5 (38)	C (38)	FLG (38)	
		TLR9 (38)	C (38)	CpG (38)	
		NOD1 (47)	C (47) / I, *Nm*, *Bb* (47)		
		NOD2 (47)	C (47) / I, *Nm*, *Bb,* LPS, FLG, CpG (47)	MDP (47)	
		RIG-I (51, 52)	C (51, 52)/I, *Nm*, *Sa*, *Sp*		
		LGP2 (51)	C (51)		
		cGAS (55)	C (55)		
		AIM2 (56, 57)	C (56, 57)		***Ba***: ↑ IL-1β (83)
		NLRP1 (56)	C (56)		
		NLRP3 (56)	C (56)		***Ba***: ↑ IL-1β (83)**LPS**: ↑ IL-1β, IL-18 (56)
		NLRC4 (56)	C (56)		

The constitutive (C) or inducible (I) expression of each PRR by human or murine microglia and astrocytes is indicated, as is the bacterial stimulus that induces expression. In addition, bacteria or their components shown to elicit glial activation and the products of such responses are indicated. *Brucella abortus* (*Ba*)*, Borrelia burgdorferi* (*Bb*), *Treponema pallidum* flagellin (FlaB3), flagellin (FLG), lipopolysaccharide (LPS), muramyl dipeptide (MDP), peptidoglycan (PGN), *Neisseria meningitidis* (*Nm*), *Staphylococcus aureus* (*Sa*), *Streptococcus pneumoniae* (*Sp*)*, Streptococcus suis* (*Ss*).

Early work established the ability of microglia (33) and astrocytes (38) to express multiple cell surface/endosomal TLRs for bacterial products, including TLR1, TLR2, TLR3, TLR4, TLR5, and TLR9 either constitutively or following activation (39–45). Subsequent studies revealed the presence of NOD2, a cytosolic NLR that senses bacterial peptidoglycan, in both microglia and astrocytes, and its upregulation upon challenge with the clinically important bacterial pathogens, *N. meningitidis* and *B. burgdorferi* (46, 47), as well as *S. suis* (48). Furthermore, murine microglia have been demonstrated to express macrophage-inducible C-type lectin receptor (Mincle), which detects glycolipids and other damage signals (49). More recently, the presence of cytosolic receptors for bacterial nucleic acids has been found in glial cells. In contrast to TLR9, an endosomal receptor for unmethylated DNA motifs that has also been reported to be expressed by astrocytes and microglia (38, 50), RIG-I has been shown to be expressed by microglia (51), mediate responses to bacterial RNA, and play a significant role in the detection of intracellular bacterial invasion by these cells (52). Similarly, microglia and astrocytes have been demonstrated to express cyclic GMP-AMP synthase (cGAS) (53–55) and AIM2 (56–59), cytosolic sensors for DNA. cGAS has recently been shown to underlie the responses of microglia to damaged self-DNA (60), raising the possibility that such DNA sensors could detect the presence of bacterial DNA in these cells, and this assertion is supported by the demonstration that *B. abortus*-induced caspase-1 activation and IL-1β release is mediated, in part, by AIM2 inflammasome activity (61). Finally, murine astrocytes have also been demonstrated to express MDA5, which has been known to detect bacterial RNA (62).

With this arsenal of PRRs, glial cells are ideally positioned to initiate acute responses to the presence of bacteria within the CNS. Importantly, the perception of bacteria and their products by glia precipitates the production of an array of inflammatory mediators, via the activation of the master inflammatory regulator NF-kB (63–70), which can then directly modulate the function of the BBB. These mediators include the key inflammatory cytokines, IL-1β, IL-6, and TNF, that are produced by glia following challenge with *N. meningitidis*, *B. burgdorferi*, *S. pneumoniae*, *S. aureus*, and *E. coli* (64, 69, 71–73). These potent inflammatory mediators have been shown to downregulate expression of critical BBB tight junction proteins, including occludin and claudin-5 (74), and this BBB disruption coincides with leukocyte recruitment to the CNS (75–77).

Consistent with this, activation of BBB endothelial cells induces the expression of adhesion molecules, such as VCAM-1, ICAM-1, and selectins, that facilitate leukocyte extravasation (78). Endothelial cell activation can occur via direct stimulation by bacteria and/or their products, indirectly due to the actions of inflammatory cytokines (79–81), or by both acting in concert (82, 83). Importantly, one study has shown that *B. abortus* can directly stimulate endothelial cells, which can be augmented by the products of glia challenged with this bacterium in such a way as to promote neutrophil transendothelial migration (83).

In addition to the production of canonical inflammatory cytokines, bacteria and their products can induce the production of matrix metalloproteinases (MMPs), reactive oxygen/nitrogen species (ROS/RNS), and perhaps excessive vascular endothelial growth factor A (VEGF-A) levels, by microglia and astrocytes, which can degrade tight junctions and basement membrane proteins thereby contributing to BBB integrity loss (84–86). For example, LPS and Gram-positive bacterial lipoteichoic acid (LTA) have been shown in induce the expression of MMPs by mixed glia, microglia, and primary astrocytes (87–90) and mediate BBB disruption (91), while *B. abortus* has been demonstrated to induce the secretion of MMP-9 by astrocytes secondary to the production of TNF (92). Similarly, soluble factors from *M. tuberculosis*-infected monocytes can stimulate astrocyte production of MMP-9 that mediates neutrophil transmigration in an *in vitro* BBB model (93). *S. pneumoniae* pneumolysin and LPS have been reported to increase intracellular ROS production by murine microglia-like cells and astrocytes (94, 95), and murine glia have been reported to show induced production of nitric oxide (NO) in response to *Streptococcus suis* (96), *S. pneumoniae* cell wall components (97), and LPS (98), although the ability of bacterial products to elicit these effects in human glial cells remains controversial (70, 99, 100). As such, the increased permeability of the BBB and cell adhesion molecule expression induced by the acute local production of inflammatory mediators by glia can function to promote the rapid recruitment of leukocytes including neutrophils into the CNS following bacterial challenge.

## 3 Glial responses to bacteria preferentially foster the rapid recruitment of neutrophils

In addition to directly or indirectly impacting the integrity of the BBB and facilitating leukocyte recruitment in general, bacterially challenged glia are a potent source of factors that favor the early entry of neutrophils into the CNS and their subsequent chemotaxis and activation. Microglia and astrocytes are well recognized to produce an array of chemokines and other chemotactic factors following activation in conditions as diverse as viral encephalitis, fungal infections, multiple sclerosis, ischemic stroke, and traumatic brain injury (101–111). Such chemotactic factors include the chemokine CCL2 that is produced by murine microglia and astrocytes in response to the fungus, *C. neoformans* (112), and mouse hepatitis virus (101), respectively, which is known to recruit multiple immune cell types including monocytes/macrophages and T cells (113), and the T-cell chemoattractant CCL20 (114, 115) that is produced by anoxic murine astrocytes (116). Similarly, both microglia and astrocytes have been demonstrated to produce an array of chemokines, including CCL2 and other T-cell recruiting chemokine, such as CXCL10, in response to amyloid-beta and/or in animal models of Alzheimer’s disease (117–121).

Importantly, bacterial components, most notably LPS, have been widely used to elicit glial activation and to induce chemoattractant mediator production (50, 87, 122–124), as shown in Table 1. This TLR4 ligand has been reported to cause the expression and/or release of the CC chemokines, CCL2, CCL3, CCL4, CCL5, CCL9, CCL20, and CCL22, and the CXC chemokines, CXCL1, CXCL2, and CXCL8, by murine and human microglia (50, 87, 124, 125). Similarly, human and murine LPS challenged astrocytes have been reported to express CCL2, CCL3, CCL4, CCL5, CCL7, CCL9, CCL12, and CCL20, and CXCL1, CXCL2, CXCL5, CXCL9, CXCL10 at the level of mRNA (126–129). However, it should be noted that inflammatory glial responses to LPS can vary according to the bacterial source. For example, murine microglial production of superoxide, MMP-9, proinflammatory cytokines and chemokines, elicited by LPS derived from *E. coli* and *M. aeruginosa,* have been shown to differ in terms of their magnitude (87).

Other bacterial components and known ligands for PRRs, in addition to LPS, have been reported to elicit chemotactic factor expression by glia, and these include bacterial flagellin, pneumococcal cell wall isolates, peptidoglycan, and the synthetic lipoprotein TLR2 agonist, PAM3CSK4 (130–134). Such stimuli have been reported to induce mRNA expression or protein release of CXCL1, CXCL2, CXCL10, CCL2, CCL3, CCL4, and CCL5, by murine microglia (130–132, 134), and CXCL2 and CCL2 by murine astrocytes (133).

In addition to studies featuring stimulation with bacterial components, multiple studies have demonstrated that both murine and human glia respond to exposure to clinically relevant intact bacteria in a manner that is consistent with an ability to recruit leukocytes to the CNS. As shown in Table 2, mixed murine glia have been demonstrated to release chemokines in response to *S. pneumoniae* (135). Consistent with this, murine and bovine microglia have been found to release CXCL2 and CXCL8, respectively, following challenge with *L. monocytogenes* (136, 137), while murine microglia also produce CXCL1 and CCL2 in response to *B. abortus* (138), CCL2, CCL3, CCL4, and CCL11 in response to *S. aureus* (139, 140), and CCL5 in response to *H. influenzae* (141). With regard to human cells, human microglia have been shown to release CXCL8 and CXCL10 in response to both *M. tuberculosis* and *B. burgdorferi* (142–144), in addition to CXCL1, CCL2, and CCL5, depending on the bacterial challenge (142–144). Similarly, human astrocytes release CCL2, CXCL1, CXCL6, CXCL8, and/or CXCL10, in response to *B. burgdorferi*, *M. tuberculosis*, and *E. coli* (142–146) and human astrocytic cells can produce CXCL3 in response to *E. coli* (147), while murine astrocytes have also been demonstrated to release CCL2 and CXCL1 in response to *B. abortus* (138), CCL2, CCL3, CCL4, and CCL11 in response to *S. aureus* (140), and CCL2 and CCL5 in response to *H. influenzae* (141).

**Table 2:** Chemokines produced by human and/or murine glia and neutrophils in response to CNS-relevant bacterial pathogens.

Cell type	Species	Stimulus	Chemokines produced
Microglia	Human	*B. burgdorferi*	**CXCL1, CXCL8**, CXCL10 (144)
		*M. tuberculosis*	*CCL2*, CCL5, **CXCL8**, CXCL10 (142)
	Murine	*B. abortus*	*CCL2*, **CXCL1** (138)
		*L. monocytogenes*	**CXCL2** (136)
		*S. aureus*	*CCL2* (139, 140), *CCL3*, CCL4, CCL11, **CXCL1** (140)
		*H. influenzae*	CCL5 (141)
	Bovine	*L. monocytogenes*	**CXCL8** (137)
Astrocytes	Human	*B. burgdorferi*	**CXCL1**, **CXCL8**, CXCL10 (143) **CXCL6** (146)
		*M. tuberculosis*	CXCL10 (142)
		*E. coli*	*CCL2*, **CXCL1, CXCL8** (145)
	Murine	*B. abortus*	*CCL2*, **CXCL1** (138)
		*H. influenzae*	*CCL2*, CCL5 (141)
		*S. aureus*	*CCL2*, *CCL3*, CCL4, CCL11, **CXCL1** (140)
Mixed Glia	Murine	*S. pneumoniae*	**CXCL2** (135)
Neutrophils	Human	*H. influenzae*	**CXCL8** (193)
		*M. tuberculosis*	**CXCL8** (175)
		*N. meningitidis* OMV	CCL4, **CXCL8** (178)
		*S. aureus*	**CXCL8** (193)
	Murine	*E. coli*	CCL2, **CXCL1** (177)
		*L. monocytogenes*	CCL2, **CXCL1** (177)
		*P. aeruginosa*	CCL2, **CXCL1** (177)
		*S. aureus*	**CXCL1** (176)
		*Y. pseudotuberculosis*	CCL2, **CXCL1** (177)

Chemokines that are recognized to preferentially recruit neutrophils are indicated in bold. OMV, Outer membrane vesicle.

While glia challenged with bacteria or their components can produce a number of chemokines, such as CCL2 and CCL3, that can promote the extravasation/recruitment of multiple leukocyte cell types (148), which may include neutrophils (149–151), a notable commonality between these stimuli is their ability to induce the expression and release of chemokines that show specificity for neutrophil recruitment. Neutrophil-linked chemokines are a distinct subgroup of these molecules that, like other chemoattractants including lipids, anaphylatoxins, and formyl peptides, show potent neutrophil attracting activity due to the limited expression of chemokine receptor types by these cells (151–153). This subgroup contains seven chemokines, CXCL1-3 and 5–8, that share a conserved ELR motif that underlies their recognition by neutrophils and their ability to recruit these cells (151–153). Importantly, significantly elevated levels of CXCL1 and CXCL8 have been detected in the CSF of human patients with meningitis associated with either Gram-negative or Gram-positive species including *N. meningitidis* and *S. pneumoniae*, respectively (154–156), and CXCL1, CXCL3, CXCL5, and CXCL8 in the CSF of human post neurosurgical bacterial meningitis patients (157).

**Figure 1:**
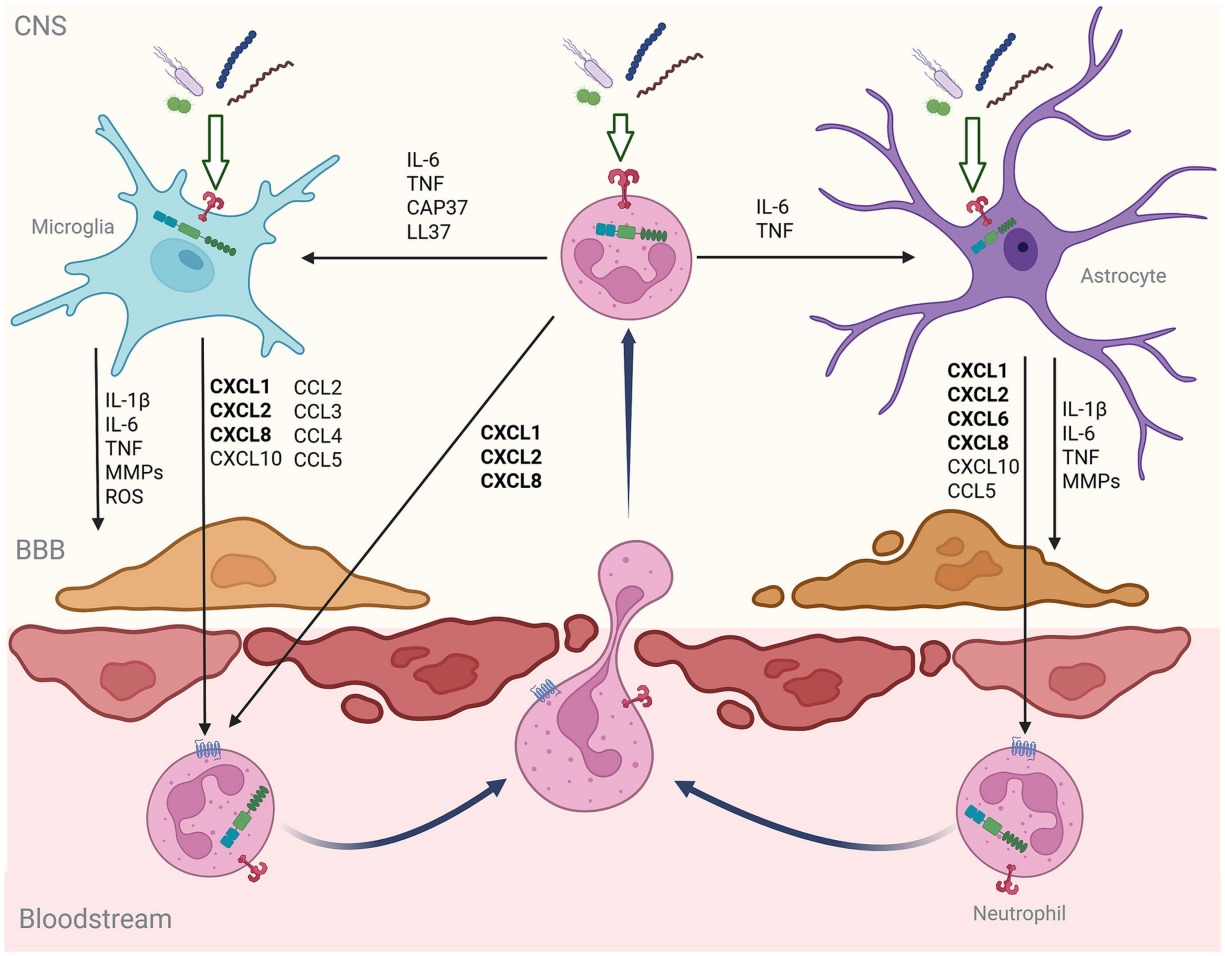
Schematic showing the proposed inflammatory glia-neutrophil axis in bacterial infections of the CNS. In this axis, microglia and astrocytes respond to clinically relevant bacteria and/or their components with a pattern of immune mediator production that promotes the recruitment of neutrophils across the blood–brain barrier (BBB) and their subsequent activation. Furthermore, neutrophils also respond to the presence of bacteria upon their arrival by producing mediators that further promote inflammatory glial functions and their own recruitment. In this manner, glia-neutrophil interactions can form a positive feedback loop that can drive damaging CNS inflammation.

Interestingly, disparate clinically relevant bacteria, including *B. abortus*, *L. monocytogenes*, *M. tuberculosis*, *S. aureus*, and *B. burgdorferi* (136–138, 140, 142, 144), and bacterial components, such as LPS (87, 125) and flagellin (134), have all been shown to induce the production of these predominantly neutrophil-recruiting chemokines by microglia. Similarly, murine and human astrocytes release CXCL1, CXCL3, CXCL6, and/or CXCL8 in response to *B. burgdorferi*, *B. abortus*, *S. aureus*, *E. coli* and bacterial components (133, 138, 143, 145–147), while mixed glial cells release CXCL2 in response to *S. pneumoniae* (135). The potential importance of this observation is underscored by reports that neutralization or inhibition of ELR motif-containing chemokine signaling reduces LPS-induced neutrophil recruitment to the CNS in mice (158, 159). Together, the common production of chemokines, including CXCL1-3 and 5–8, by both microglia and astrocytes in response to diverse bacterial challenges, implicates these cells in the rapid and preferential recruitment of neutrophils following bacterial CNS infection (as shown schematically in Figure 1).

Despite this body of evidence supporting a central role for resident glial cells in the early recruitment of neutrophils following bacterial invasion of the CNS, it should be noted that many studies have relied upon high-throughput analytical approaches that have exclusively assessed expression of chemotactic factors at the level of mRNA without confirmation of protein production. In addition, bacterial components such as LPS have often been employed as a stimulus rather than whole organisms, and this reductionist approach may not accurately model host cell responses to complex bacterial stimuli that will likely be perceived by multiple cytosolic and cell surface receptors generating an integrated cellular response. Furthermore, the clinical significance of findings in murine glial cells to organisms such as *H. influenzae* have not been confirmed in authentic human cells (as highlighted in Table 2), while information regarding some of the most clinically important bacterial species such as *N. meningiditis*, *S. pneumoniae*, and *L. monocytogenes*, is lacking.

## 4 Neutrophils respond to bacteria upon CNS entry to promote their further recruitment

The rapid recruitment of neutrophils into the CNS is a common feature of many insults. In animal models of ischemic stroke and traumatic brain injury, neutrophils have been found to infiltrate the subdural and subarachnoid spaces and brain parenchyma within hours (160, 161). Peripheral inflammation resulting from systemic bacterial LPS administration has been shown to promote neutrophil recruitment to the brain parenchyma in mice (162), while elevated numbers of these cells have been detected in the CSF of bacterial meningitis patients as early as 12 h following the onset of symptoms (163). Upon recruitment to the CNS, neutrophils can combat bacteria directly by phagocytosis, the release of antimicrobial agents such as ROS (27, 164–166), and the production of neutrophil extracellular traps (NETs) (167), as evidenced by their detection in the CSF of pneumococcal meningitis and Lyme neuroborreliosis patients (168).

Neutrophils can perceive bacterial components, such as LPS, lipoprotein, and DNA, via PRRs to upregulate the expression of cell surface molecules, including the CD11b/CD18 complex (169, 170), and potentiate their ability to migrate toward chemoattractants (171). Furthermore, such PRR-mediated neutrophil activation induces the expression and/or release of the signature proinflammatory cytokines, TNF, IL-6, and IL-1β (170, 172, 173) and promotes their survival by delaying apoptosis (174). Importantly, bacterial pathogens of the CNS, including *M. tuberculosis*, *S. aureus*, *E. coli*, and *L. monocytogenes*, have been shown to elicit inflammatory responses by murine or human neutrophils (175–177), and outer membrane vesicles from Group B *N. meningitidis* has been found to increase the expression and/or release of TNF and IL-1β by human neutrophils (178), as summarized in Table 1.

Furthermore, recruited neutrophils can also perceive and respond to damage associated molecular patterns (DAMPs) that are produced locally within the CNS as a direct consequence of bacterial challenge or secondary to the effects of acute inflammation. For example, neutrophils are known to respond to extracellular ATP, mitochondrial DNA, heat/cold shock proteins, and HMGB1, in a manner that promotes their migration and/or production of ROS and neutrophil-recruiting chemokines (179, 180). Importantly, many of these DAMPs have been shown to be released by glial cells in response to inflammatory mediators (181). Furthermore, some CNS bacterial pathogens, including virulent strains of *S. aureus*, can induce neutrophil lysis via necrosis or necroptosis, which may result in their own release of DAMPs (182).

Neutrophil-derived IL-1, IL-6, and TNF would then be anticipated to further impact BBB integrity and drive brain endothelial cell adhesion molecule expression to augment neutrophil extravasation in a positive feedback manner (74). In addition, bacteria product and inflammatory cytokine-induced neutrophil degranulation and/or NET formation will result in the release of factors, including cathepsin G, elastase, and proteinase 3, which can all degrade extracellular matrix components and increase endothelial cell layer permeability (167, 183–188). Furthermore, ROS originating from neutrophils, shown to be released in response to the relevant CNS pathogens, *S. pneumoniae* (189), *L. monocytogenes*, *S. aureus*, and *P. aeruginosa* (190), will also contribute to BBB degradation. Finally, bacterial products can induce neutrophil microvesicle release that can be internalized by human brain endothelial cells, causing dysregulation in the expression of genes associated with tight junction formation and resulting in increased monolayer permeability (191).

Importantly, in addition to their effects on BBB that can foster further leukocyte extravasation, neutrophils can respond to bacteria and their components in a manner that can directly promote further neutrophil recruitment. Human neutrophils respond to LPS, single stranded bacterial DNA, and *N. meningitidis* outer membrane vesicles, with the production of the potent neutrophil chemoattactant, CXCL8, and other chemokines capable of potentiating neutrophil migration, including CCL3 (170, 173, 178, 192). More importantly, the CNS pathogens, *M. tuberculosis*, *L. monocytogenes*, *S. aureus*, *E. coli*, *H. influenzae*, and *P. aeruginosa*, have all been shown to induce the expression of the neutrophil attracting chemokines CXCL1 and CXCL8 in murine or human neutrophils (175–177, 193), as summarized in Table 2.

In addition, neutrophils entering the CNS can also promote their further recruitment indirectly by modulating the immune functions of glia (194). The potential importance of such a positive feedback loop within the CNS has been shown in mouse models of Alzheimer’s disease where both LFA1 blockade and neutrophil depletion results in reduced microgliosis (195), and knockout of the NET-associated signaling component, PAD4, leads to reduced glial cell activation following CNS bacterial endotoxin challenge (196). Products of activated neutrophils, such as IL-1β, IL-6, TNF, LL37, and CAP37, have been demonstrated to exert priming effects on microglia and/or astrocytes that include increased phagocytosis and MHC class II molecule expression, induced migration, augmented inflammatory cytokine production, and the production of the BBB-modulating products VEGF and vimentin (197–204). In addition to their secreted products, neutrophils have also been shown to make direct physical contact with microglial processes in an endotoxin challenge model suggesting a potential role for cell–cell crosstalk between resident CNS cells and recruited neutrophils in acute CNS inflammation (162), while neutrophils have been found to elicit phenotypic changes in murine astrocytes in *in vitro* co-culture studies (205, 206).

Perhaps more intriguingly, neutrophil products may impact glial immune functions in such a way as to specifically promote further neutrophil recruitment. For example, neutrophil-derived myeloid-related protein 14 (MRP14) is released by human neutrophils following bacterial challenge (207) and this neutrophil product has been found to promote microglial pyroptosis in a mouse model of acute ischemic stroke that subsequently facilitates neutrophil mobilization from the bone marrow (208). In addition, the combined stimulation of human astrocytes with IL-1β and TNF, which are both produced by activated neutrophils, can induce the elevated expression of the neutrophil-recruiting chemokines, CXCL1 and CXCL8, and factors that can promote the recruitment of leukocytes including neutrophils, CCL3 and C5a, by these cells (204).

Together, the available evidence indicates neutrophils cross the BBB following bacterial infection and can respond to CNS-relevant bacterial pathogens, their products, and/or local DAMPs, in a manner that can contribute to neuroinflammation. Furthermore, such responses can facilitate their further recruitment to the CNS by additionally altering the permissiveness of the BBB for neutrophil extravasation and migration either directly or indirectly via the modulation of the immune functions of microglia and astrocytes. Such a positive feedback loop, illustrated in Figure 1, could serve to drive further neuroinflammation with potentially devastating consequences.

However, it should be noted that evidence definitively establishing a central role for such a self-perpetuating loop in bacteria-induced neuroinflammation is lacking. For example, the neutrophil-recruiting responses of glia to many clinically important bacterial CNS pathogens, such as *N. meningitidis* and *S. pneumoniae*, have been understudied. To bridge this knowledge gap, studies demonstrating protein production, rather than mRNA expression, of neutrophil-attracting chemokines by glia in response to intact viable bacteria are required. Similarly, there has been little analysis of neutrophil-mediated effects on glial functions in the context of bacterial challenge. Approaches including the application of neutrophil-derived conditioned media following bacterial challenge to glial cells could shed light on such interactions. This dearth of knowledge is especially acute for glia-neutrophil interactions that occur during *in vivo* infection. While in vivo microglial depletion has recently been shown to reduce inflammatory cytokine levels in the brain in a systemic infection mouse model of pneumococcal meningitis and increase survival (Farmen et al., Submitted), relatively few studies have examined glial responses to bacteria *in situ*. This is due, in part, to the paucity of animal models that faithfully reproduce human CNS infections. For example, systemic LPS administration has been employed as a surrogate for bacterial infection, but shows poor CNS penetrance (209), is typically administered only once, which may not result in glial cell activation (210), elicits variable effects depending on the route of administration (211), and is often derived from bacterial species that are not typically associated with CNS disease (211, 212). Regarding whole organisms, many bacterial species associated with human infection, including *B. burgdorferi*, *E. coli*, *N. meningitidis*, and *M. tuberculosis*, either fail, or show limited ability, to breach the BBB in most laboratory animal models, sometimes requiring co-morbidities or physical trauma to enter the CNS (213–217).

Furthermore, some of the studies conducted have yielded contradictory findings with neutrophils being shown to be both protective (218) and damaging (219) in murine models of pneumococcal meningitis. Finally, there has been no definitive demonstration of the preferential recruitment of neutrophils by glia by approaches such as the conditional knockout of glia produced neutrophil-recruiting chemokines.

## 5 Glial responses adjust temporally in a manner that could protect against neutrophil-mediated inflammatory damage

**Figure 2:**
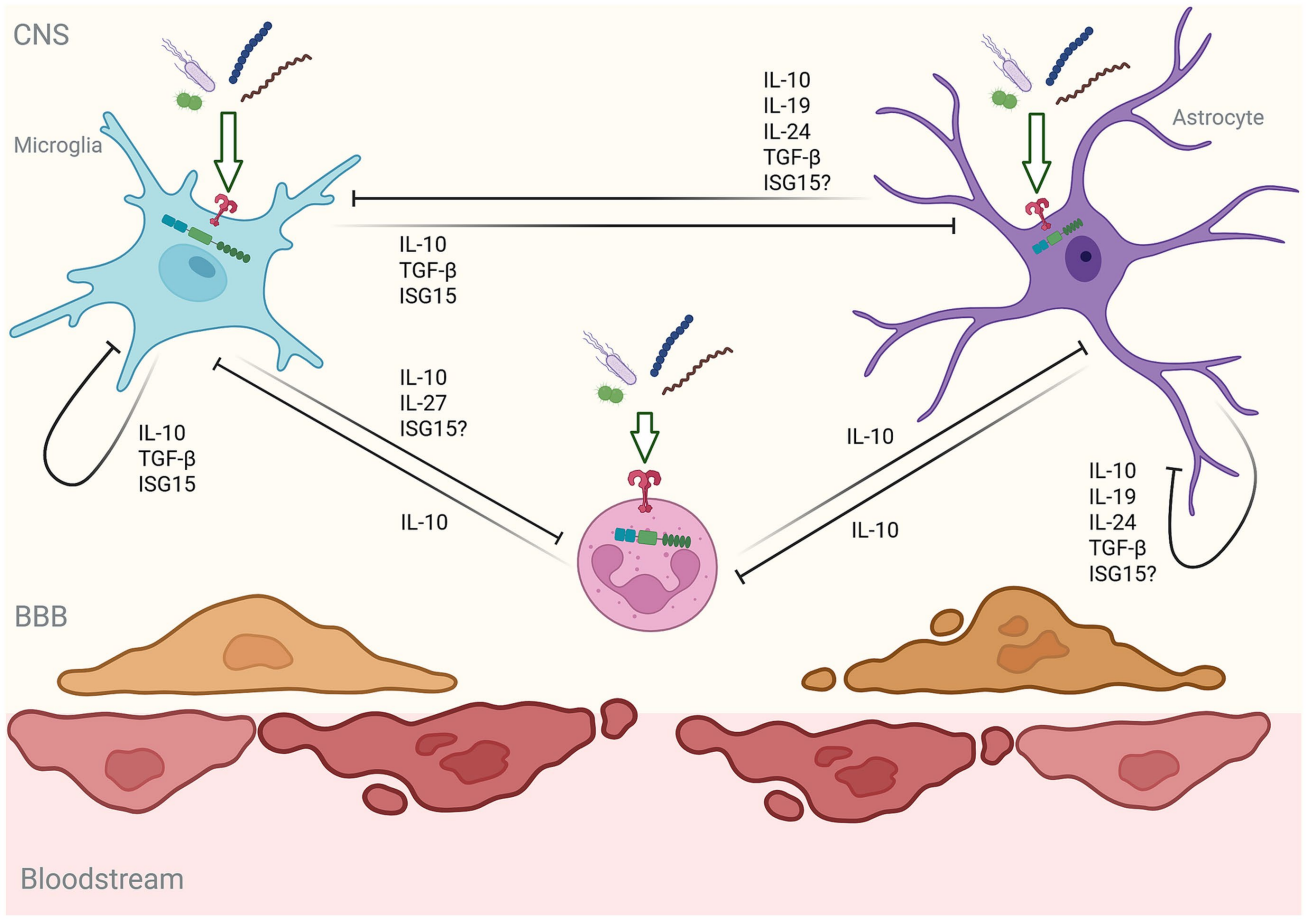
Schematic showing how a compensatory anti-inflammatory response by glia and neutrophils might limit bacteria-induced neuroinflammation. Secondary to an acute inflammatory response induced by bacteria, glia and perhaps neutrophils show chronicity in their immune mediator production. Bacterially challenged glia release a variety of anti-inflammatory mediators in a delayed manner that can attenuate their inflammatory functions or reduce oxidative burst potential, phagocytic activity, cell survival, and inflammatory cytokine release by neutrophils. Similarly, neutrophils in the CNS have the potential to release anti-inflammatory mediators that could attenuate the pro-inflammatory responses of glia.

Clearly, glial cells can respond to bacterial stimuli in a manner that can initiate and/or potentiate acute inflammation within the CNS. However, it is becoming increasingly apparent that glial responses to such challenges temporally change in a similar manner to that seen in the periphery, with the swift but transient release of inflammatory mediators giving way to the production of factors that can limit damage and promote inflammation resolution (Figure 2). Of such immunosuppressive factors, IL-10 and related cytokines are perhaps the best characterized (220, 221). Systemic administration of LPS has been shown to elicit rapid induction of mRNA encoding inflammatory cytokines in the CNS but this shifts to the elevated expression of immunosuppressive cytokines, including IL-10, by 24 h following challenge (222). Importantly, microglia and astrocytes have been reported to produce members of the IL-10 family of cytokines in a delayed manner (223). Indeed, *B. burgdorferi* protein NapA has been shown to stimulate IL-10 production by human microglia (224), while *N. meningitidis* and *B. burgdorferi* lysates elicit the robust production of IL-10 by murine microglia and astrocytes, but only at 24 h following challenge (225). This delayed onset appears to occur secondary to the autocrine/paracrine actions of inflammatory mediators, as conditioned media from bacterially challenged glia could elicit IL-10 production from unstimulated cells (225). Such a finding is consistent with prior demonstrations that microglia produce IL-10 in response to IL-6 and TNF (223, 226).

Glia have also been shown to produce other members of the IL-10 cytokine family in response to exposure to bacteria or their components, which have immunosuppressive activity. For example, murine and human astrocytes, but not microglia, produce IL-19 in response to *N. meningitidis* or bacterial LPS and flagellin in a delayed manner (24 h), and such findings are consistent with the observation that levels of mRNA encoding IL-19 are significantly increased in the CNS of mice at 72 h following infection with *S. pneumoniae* (123). Similarly, mRNA encoding another immunosuppressive IL-10 family member, IL-24, was also increased in the brain of *S. pneumoniae*-infected mice at this time point (227), and isolated murine astrocytes were found to produce IL-24 in response to bacterial LPS or *N. meningitidis* (227).

In addition to IL-10 family members, glia can express the immunosuppressive cytokine TGF-*β*. Acute exposure (1–6 h) to LPS or heat-killed *Listeria monocytogenes* has been shown to reduce constitutive TGF-β mRNA expression in primary rat microglia (228), consistent with the early proinflammatory response of these cells to bacterial challenge. Interestingly, microglia have been shown to produce elevated levels of TGF-β in response to *B. burgdorferi* protein NapA (224), or LPS and proinflammatory cytokines but do so at 48 h following challenge (229). Furthermore, astrocytes may also be a source of this anti-inflammatory cytokine secondary to the actions of IL-10 (230). As such, glia may produce TGF-β as an additional means to suppress sustained inflammation associated with bacterial infection, but it should be noted that the relevance of such responses to whole organism bacterial challenge has not yet been demonstrated.

Finally, glia may produce additional factors that can serve a protective function against inflammatory damage associated with bacterial infection. For example, glia express excitatory amino acid transporters (EAATs), which combat excessive neuron excitotoxicity (231), and such expression is elevated in glia in response to bacterial LPS, proinflammatory cytokines, and pleiotropic cytokines like IL-24 (227, 232). Similarly, murine microglia can produce IL-27, a cytokine that has classically been reported to exert anti-inflammatory effects, in response to LPS (233). Also, endotoxin challenge *in vivo* can induce the robust late expression of the TH2 cytokine IL-13 by glia (234), which has been reported to have neuroprotective effects and resolve inflammation (235). However, evidence for such responses for intact whole organism bacteria is again lacking.

Most, if not all, these immunosuppressive mediators have the potential to act in an autocrine/paracrine manner to suppress the inflammatory responses of glial cells. IL-10 can act via STAT3 to inhibit NF-κB-mediated inflammatory gene expression in microglia (236) and such responses appear to be beneficial as demonstrated by the protection conferred by conditional deletion of the IL-10 receptor in microglia following systemic LPS challenge (237). With regard to other IL-10 family members, cultured microglia and astrocytes express the receptors for IL-19 and IL-24, either constitutively or inducibly following bacterial challenge, and these cytokines can induce mRNA expression of the negative regulator of cytokine signaling, SOCS3, while markedly attenuating glial IL-6 and TNF-*α* responses to *N. meningitidis*, *S. pneumoniae*, or bacterial components (123, 227). Lastly, TGF-*β* receptor subunit deletion appears to increase microglial inflammatory mediator expression, supporting a suppressive effect of this cytokine on glial immune functions (238).

The ubiquitin-like protein, ISG15, has been recognized to mediate antiviral immunity (239), but it has also been shown to exert anti-inflammatory effects (240) and to be produced by leukocytes, including granulocytes, in response to bacterial LPS or *M. tuberculosis* (241, 242). Recently, we have determined that a human microglial cell line can express and release ISG15 in response to LPS and a clinical strain of *N. meningitidis* and lipooligosaccharides isolated from this organism (Dunphy et al., Submitted). Interestingly, endogenous ISG15 has been shown to prevent TLR4-mediated NF-κB activation and associated inflammatory cytokine production by human microglia-like cells (243), and we have found that exogenous recombinant ISG15 can similarly inhibit *N. meningitidis*-induced NF-κB activation and inflammatory mediator production in such cells (Dunphy et al., Submitted). These observations may be indictive of a novel negative feedback loop, whereby the recognition of bacterial motifs precipitates ISG15 expression by resident microglia that subsequently mitigates further neuroinflammatory responses.

Importantly, glia-derived suppressive cytokines also have the potential to attenuate neutrophil-mediated inflammatory and/or antimicrobial responses, survival, and further recruitment. IL-10 is well known to attenuate neutrophil phagocytosis and bacterial killing (244, 245), oxidative bursts (246, 247), inflammatory cytokine release, and chemokine production (248). Furthermore, IL-10 can negatively impact bacterial component-induced increases in neutrophil longevity (249). Similarly, IL-27 can suppress bacterial component-induced human neutrophil adhesion and ROS and cytotoxic granule component release (250), while IL-19 can block neutrophil activation (251).

Also, neutrophils themselves can be a source of immunosuppressive cytokines in response to bacteria or their components. For example, they can produce IL-10 following challenge with LPS, or mycobacteria, *E. coli*, and *S. flexneri* antigens (252, 253), and IL-1RA in response to pro-inflammatory cytokines (254). Neutrophils are known to contribute to their own clearance upon engagement with bacteria or their components at sites other than the CNS, where neutrophils generate “find me” and “eat me” signals to promote efferocytosis and macrophage repolarization (255). Furthermore, neutrophils may undergo apoptosis following phagocytosis of bacterial species associated with CNS infection including *E. coli* (256, 257), *M. tuberculosis* (258), *S. pneumoniae* (259), *S. aureus* (260), and *L. monocytogenes* (261). Although, it should be noted that such neutrophil responses have not specifically been demonstrated to occur in the CNS following bacterial infection. As such, apoptotic neutrophil death has the potential to serve a protective function in the brain by eliminating a key cellular component in the inflammatory response.

Together, the shift to the production of anti-inflammatory/immunosuppressive factors by glial cells and/or recruited neutrophils, and the induced death of neutrophils that have entered the CNS, may all attenuate excessive and/or prolonged neuroinflammation to mitigate the inflammatory damage associated with bacterial infection of the CNS. However, the generation of such compensatory responses could compromise the host’s ability to achieve sterilizing immunity. In support of this notion, TGF-*β*-mediated signaling has been associated with decreased *S. pneumoniae* clearance (262), while IL-10 can reduce neutrophil bactericidal/phagocytic activity (263). A failure to clear bacteria may then lead to “smoldering” or relapsing infections that continue to result in direct damage to the CNS. Indeed, up to 6% of meningitis cases associated with bacteria, including *S. pneumoniae* and *H. influenzae*, have been reported to be relapsing (264) and can occur days, or even years, later (265). Alternatively, such persistent infections can serve as a trigger for the development of other chronic neuroinflammatory disorders.

## 6 Bacteria-induced glial and neutrophil-mediated responses could promote chronic neuroinflammatory disorders

While prophylactic or therapeutic interventions can be effective in preventing lethal outcomes following bacterial CNS infection, it is well recognized that the lingering presence of PAMPs, DAMPs, and/or inflammatory effector molecules can cause further/sustained damage to the CNS (266, 267). For example, the BBB may stay permeable for weeks, months, or even years, after a traumatic event has been resolved (268, 269) and, while bacterial infections trigger acute BBB destabilization, there is evidence in animal models of sepsis meningitis that this effect may be long lasting (6, 270, 271). Levels of inflammatory cytokines in the CNS remain elevated in mouse models of systemic bacterial infection and endotoxic shock, and glial populations in these conditions may acquire a primed state wherein they demonstrate increased sensitivity to subsequent insults (272–274).

Neutrophils are a critical source of MMPs (275, 276) and chronic release of MMP2, MMP8, and MMP9 have been observed in several CNS disorders, including Parkinson’s and Alzheimer’s diseases, but have also been seen with bacterial stimuli (84, 275). Such sustained production of MMPs can contribute to BBB instability by degrading tight junctions and basement membrane proteins (84, 277). However, further studies are required to definitively link neutrophil-mediated MMP production to sustained BBB compromise following CNS infection. Similarly, cathepsins can degrade extracellular matrix components and are released by microglia (278–281) and neutrophils (186) in response to bacterial LPS, and elevated levels of these molecules has been associated with microglial polarization to an inflammatory and/or neurotoxic phenotype (278, 279). Increased levels of cathepsin have been associated with chronic neuroinflammatory conditions (281–283) but, again, the link between glial and/or neutrophil-mediated cathepsin production and sustained BBB compromise following bacterial infection of the CNS has yet to be established.

There is also mounting evidence that many clinically important bacterial pathogens can invade and persist in either glia or neutrophils. For example, Group B Streptococcus and *S. aureus* can enter and survive in astrocytes for up to 24 h (140, 284), while encapsulated *S. pneumoniae*, *S. aureus* and *C. koseri* demonstrate intracellular survival in microglia (140, 285, 286). *S. aureus* appears to subvert both autophagy and apoptosis in human neutrophils to maintain intracellular survival (287), and *M. tuberculosis*, *L. monocytogenes*, and some *E. coli* strains can evade internal neutrophil oxidative burst killing (288–290). In addition, anti-inflammatory mediators, such as IL-19, can reduce neutrophil apoptosis (291) and such an effect might further promote intracellular bacterial survival.

Finally, in addition to bacterial persistence and associated ongoing glial stimulation, even a transient neuroinflammatory response may result in the sensitization of microglia and astrocytes to subsequent stimulation (292–294). Such “priming” has been suggested to play a role in the development of long-term neurodegenerative disorders (295). For example, neuroinflammation induced peripherally following LPS administration is associated with microglia and astrocyte activation, which then results in the increased presence of products, such as amyloid-beta plaques, that have been linked to neurodegenerative diseases including Alzheimer’s disease (210).

## 7 Conclusion: the glia-neutrophil axis represents a potential target for therapeutic intervention to limit inflammatory damage associated with bacterial infection of the CNS

Bacterial infections of the CNS are often associated with rapid and devastating neuroinflammation. The bacteria responsible for these infections demonstrate the ability to breach the anatomical barriers, such as the BBB, which protect the CNS. While inflammation plays an important physiological role in defense against bacterial pathogens, these responses within the confines of the cranium can lead to irreversible neuronal damage, long-term sequelae, or death. Here, we have described the ability of resident glial cells including microglia and astrocytes, to perceive clinically relevant bacteria and their products via various PRRs present on their cell surface or within the cytosol. These glial cells then respond in a manner that can promote inflammation, changes to BBB integrity, and recruit leukocytes into the CNS. In this review, we have summarized their ability to produce chemotactic factors in response to bacterial components such as LPS, peptidoglycan, and flagellin, and bacterial CNS pathogens that include *B. burgdorferi*, *S. aureus*, and *S. pneumoniae*. Importantly, we have highlighted the fact that the chemotactic factors produced by bacterially challenged glial cells tend to preferentially recruit neutrophils, and we have described how these cells could then respond to the presence of bacteria to further promote glial activation and their own recruitment. This then, could form a self-perpetuating cycle that precipitates the rapid inflammatory CNS damage associated with bacterial infection. Furthermore, these responses may result in the priming of glial cells that has been suggested to underlie or facilitate the development of chronic neuroinflammatory disorders in those that survive an acute inflammatory insult. As such, the presence of elevated levels of such neutrophil chemoattractants in the CSF could be leveraged as a novel early biomarker for neurological disease severity risk. Furthermore, the disruption of this vicious cycle could represent an attractive target for new adjunctive therapeutic interventions that could be employed early upon symptom manifestation to limit CNS inflammation progression.

However, it is also becoming apparent that glia, and perhaps neutrophils themselves, can adjust their responses to bacterial challenge temporally in such a way as to protect against damage mediated by this glia-neutrophil axis. Here, we have described the available evidence for the delayed production for anti-inflammatory mediators, including IL-10, IL-19, and TGF-*β*, by glial cells in response to clinically relevant bacteria. Similarly, neutrophils can also produce IL-10 in response to bacteria and augment their own clearance by promoting efferocytosis. If such a temporal shift is indeed an attempt by the host to interrupt runaway CNS inflammation, the promotion of this compensatory mechanism might also be targeted therapeutically to protect against infection. However, it should be noted that this approach would run the risk of preventing sterilizing immunity that could result in persistent infections and/or the development of chronic neurodegenerative disorders.

While we have presented the available evidence to support a role for a glia-neutrophil axis in bacterial CNS infections, it is important to note the numerous caveats and knowledge gaps that exist in our understanding of such interactions. For example, many studies in this area have relied on the analysis of changes in mRNA expression by host cells and lack confirmation at the protein level, while others have restricted their investigations to the use of bacterial components rather than whole organism clinically relevant bacteria. Furthermore, most of these *in vitro* reports feature the use of murine cells and/or immortalized cell lines rather than the study of authentic primary human glia and neutrophils. Critically, these in vitro experiments also lack the cell–cell interactions that would be anticipated to occur *in situ* between glial cell types and infiltrating leukocytes during bacterial infection of the CNS, and there is a currently a dearth of *in vivo* analysis of them. This is largely due to a lack of *in vivo* animal models that faithfully reproduce human disease, with researchers resorting to direct bacterial administration into the CNS, systemic endotoxin administration, or even cecal ligation and puncture to evoke CNS neuroinflammation. Clearly, more physiologically accurate animal models of bacterial CNS infection are needed that would, in combination with the conditional cell type specific knockout of select immune mediators, allow the detailed description of such a glia-neutrophil axis and its possible manipulation for therapeutic purposes.
